# The evidence base for physiotherapy in myalgic encephalomyelitis/chronic fatigue syndrome when considering post-exertional malaise: a systematic review and narrative synthesis

**DOI:** 10.1186/s12967-020-02683-4

**Published:** 2021-01-04

**Authors:** Marjon E. A. Wormgoor, Sanne C. Rodenburg

**Affiliations:** 1 Division of Mental Health and Addiction, Vestfold Hospital Trust, Tønsberg, Norway; 2 Division Physical Medicine and Rehabilitation, Vestfold Hospital Trust, Stavern, Norway; 3 Department of Physiotherapy, Hanze University of Applied Sciences Groningen, Groningen, The Netherlands

**Keywords:** Myalgic encephalomyelitis, Chronic fatigue syndrome, Post-exertional malaise, Physiotherapy, Intervention, Systematic review

## Abstract

**Background:**

Due to the inconsistent use of diagnostic criteria in myalgic encephalomyelitis/chronic fatigue syndrome (ME/CFS), it is unsure whether physiotherapeutic management regarded effective in ME/CFS is appropriate for patients diagnosed with criteria that consider post-exertional malaise (PEM) as a hallmark feature.

**Purpose:**

To appraise current evidence of the effects of physiotherapy on symptoms and functioning in ME/CFS patients in view of the significance of PEM in the applied diagnostic criteria for inclusion.

**Methods:**

A systematic review of randomized controlled trials published over the last two decades was conducted. Studies evaluating physiotherapeutic interventions for adult ME/CFS patients were included. The diagnostic criteria sets were classified into three groups according to the extent to which the importance of PEM was emphasized: chronic fatigue (CF; PEM not mentioned as a criterion), CFS (PEM included as an optional or minor criterion) or ME (PEM is a required symptom). The main results of included studies were synthesized in relation to the classification of the applied diagnostic criteria. In addition, special attention was given to the tolerability of the interventions.

**Results:**

Eighteen RCTs were included in the systematic review: three RCTs with CF patients, 14 RCTs with CFS patients and one RCT covering ME patients with PEM. Intervention effects, if any, seemed to disappear with more narrow case definitions, increasing objectivity of the outcome measures and longer follow-up.

**Conclusion:**

Currently, there is no scientific evidence when it comes to effective physiotherapy for ME patients. Applying treatment that seems effective for CF or CFS patients may have adverse consequences for ME patients and should be avoided.

## Background

Myalgic encephalomyelitis (ME)/chronic fatigue syndrome (CFS) is a serious long-term, multi-system disease. It is characterized by severe unexplained fatigue that is not improved by rest and is accompanied by symptoms related to cognitive, immune and autonomous dysfunction [1, 2]. Disease severity varies from mild (50% reduction of premorbid activity level) to very severe (completely dependent and bedridden).The recovery rate seems generally poor and most patients never regain their pre-disease level of health [3].

Previously, and still by some clinicians and research groups, ME/CFS was understood and approached by applying a psychogenic or psychosomatic model [4, 5]. Onset and continuance of the illness were then considered to be perpetuated by patients’ irrational beliefs, avoidance behavior, health anxiety, hypochondriasis or personality traits. Yet, although the exact cause of ME/CFS is still unknown, there is generally consensus on a biomedical understanding [1, 6]. A number of studies demonstrated multiple pathophysiological disturbances mostly comprised of changes in neurological, immunological, metabolic, endocrinological and cognitive functioning [1, 2, 6–8].

A considerable amount of the patients diagnosed with ME/CFS show prolonged exacerbation of their symptoms after minimal amounts of physical, sensory, emotional or cognitive effort [1, 9–11]. This phenomenon is termed post‐exertional malaise (PEM). Its onset is often delayed and has an unpredictable recovery period that may last days, weeks or even months. The severity and duration of symptoms are out of proportion to the exertion [1, 12]. Patients tend to describe PEM as the most debilitating part of the disease [13]. PEM is not synonymous with post-exertional fatigue, not explained by deconditioning or malingering and is rarely found in other fatiguing illnesses [1, 10]. Hence, patients’ reduction in activity should not be understood as ‘fear avoidance behavior’, but rather as a rational and physical response to the occurrence of PEM [4, 14]. Various biomedical and provocation studies have confirmed this abnormal response to exertion [1, 2, 15–21].

In the absence of valid diagnostic tests, ME/CFS is diagnosed with clinical criteria when alternative diagnoses are excluded. In line with the different perspective of explanatory models of pathogenesis and pathophysiology, over 20 different diagnostic criteria sets have been created for research and clinical purposes. PEM is included in several of the diagnostic criteria, although there are different views on its significance in the diagnosis of ME/CFS. The broadest criteria set, Oxford [22], is unspecific and only requires severe, disabling fatigue, affecting physical and mental functioning for a minimum of six months and does not consider PEM at all. Other criteria sets include PEM as an optional symptom (e.g. CDC-94/Fukuda criteria [23]) and require the presence of more symptoms. The Fukuda criteria are the most frequently applied diagnostic criteria in current research. The 2003 Canadian Consensus Criteria (CCC) [24], the newer International Consensus Criteria for ME (ME-ICC) [25] and Systemic Exertion Intolerance Disease criteria (SEID) [1] require the presence of PEM, substantial impaired function and other core symptoms including pain, unrefreshing sleep, cognitive impairment, orthostatic intolerance or neuroendocrine dysfunction [26]. Consequently, these narrow criteria sets create a more homogenous patient group with a higher symptom burden and far higher levels of physical and cognitive disability than broader criteria [27, 28]. Broad diagnostic criteria may also embrace people who may have a form of chronic fatigue that, in many cases, primarily involves psychological factors [29].

Several different names have been proposed for this disease. The most commonly used are “Myalgic Encephalomyelitis”, “Chronic Fatigue Syndrome”, and the umbrella-term ME/CFS. Whether PEM is a cardinal feature of ME/CFS, and accordingly whether ME and CFS are distinct clinical entities, has been debated for almost two decades [30]. For purposes of clarity, in this review, the label “ME” will be used when PEM is a cardinal feature and the other core symptoms are present as well [31]. “CFS” will be used when PEM or other core symptoms are optional features. The label “Chronic Fatigue” (CF) will be applied when PEM is not accounted for at all. When discussing ME, CFS and/or CF in general, the umbrella-term “ME/CFS” will be pragmatically applied in this review.

Physiotherapists are often involved in the management of ME/CFS patients [32]. In the last decade, several systematic reviews and meta analyses that included interventions that seem relevant for physiotherapeutic management of adult ME/CFS patients have been published [29, 33–46]. However, generally, the applied diagnostic criteria were not explicitly accounted for in these reviews. Patients diagnosed with different criteria may have different symptoms as well as reactions to certain interventions, leading to the diagnostic incongruences and treatment challenges seen in ME/CFS.

In Europe, few countries have guidelines for the clinical approach to ME/CFS [47]. Typically, it is not clear which diagnostic criteria the recommendations for illness management are based on, or who the target population is. Despite this, the recommendations mainly consist of cognitive behavioral therapy (CBT) and graded exercise therapy (GET) [47]. It is not well documented how these recommended clinical interventions affect patients with ME, but they are criticized by clinicians, patients and researchers as being inappropriate for patients with PEM [19, 48]. The evidence of the effect of commonly applied ME interventions is currently of increased relevance due to possible consequences of the ongoing COVID-19 pandemic*.* ME/CFS has been linked to many different viruses. Experiences from past epidemics and current observations suggest that a considerable number of patients recovering from COVID-19 may develop ME/CFS-like symptoms [49].

The aim of this review was to appraise current evidence of effects of physiotherapy on symptoms and functioning in ME/CFS patients in light of the significance of PEM in the applied diagnostic criteria for inclusion. The objectives were:

- To summarize current evidence of the effects of physiotherapeutic interventions on symptoms and functioning in ME/CFS patients.
- To synthesize the findings in light of the significance of PEM in the applied diagnostic criteria for inclusion.
- To evaluate and discuss the reported physiotherapeutic interventions in view of (potential) harm and adverse effects for patients with ME.

## Methods

### Design

A systematic review methodology was utilized to evaluate benefits and potential harms and adverse events of applied physiotherapeutic interventions in ME/CFS patients. The studies were grouped and evaluated according to the diagnostic criteria used. The review was limited to randomized controlled trials (RCTs).

### Search strategy

**Fig. 1:**
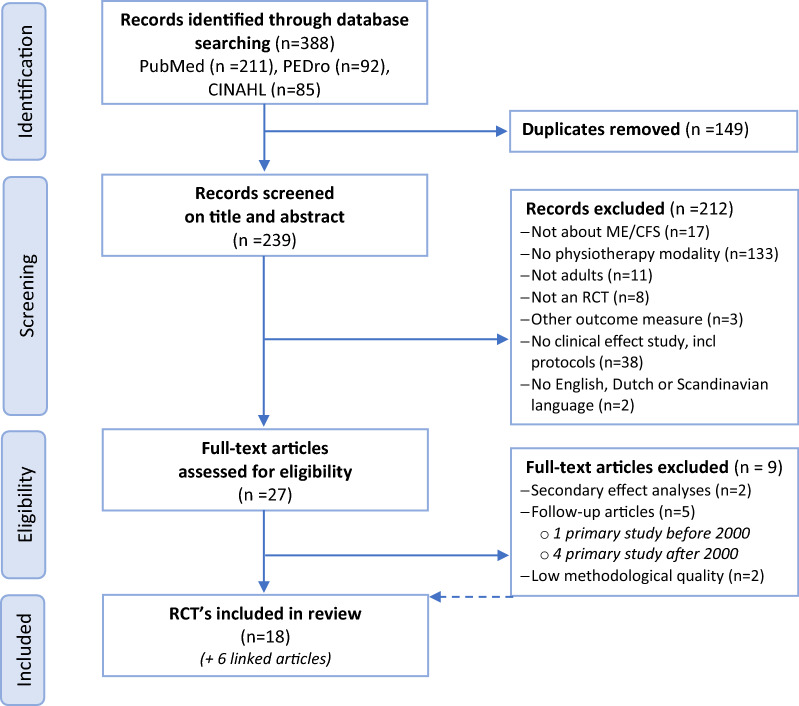
Flowchart of study selection

The systematic search for relevant RCTs was conducted according to the Preferred Reporting Items for Systematic Reviews and Meta-analysis (PRISMA) guidelines [50] (see Fig. 1). PubMed, CINAHL and PEDro were searched with the following search words in the title: myalgic encephalomyelitis, chronic fatigue syndrome, CFS, chronic fatigue, post-exertional neuroimmune exhaustion, PENE, systemic exertion intolerance disease or SEID. The search was filtered to RCTs published since the year 2000. This literature search was undertaken and reviewed by the second author between February and April 2020 and subsequently repeated by both authors.

### Eligibility criteria

Articles were screened with the following inclusion criteria: (1) RCT, (2) population of patients diagnosed with CFS and/or ME, (3) an evaluation of the efficacy of a physiotherapeutic intervention (i.e. physical activity/exercise therapies, manual therapies, body awareness, electrotherapy techniques or health education), (4) outcome measures evaluating physical or mental symptoms and/or functioning, including quality of life.

Articles were excluded when they (1) were not available in full text, (2) were not written in English, Dutch or a Scandinavian language, (3) had an exclusive underage patient population, (4) had a follow-up article of a primary study published before 2000 or (5) had a poor methodological quality (PEDro score 0–3).

### Methodological quality analysis

The PEDro scale was used in order to evaluate the methodological quality of the RCTs [51]. This is a scale with 11 items, on which a “1” or a “0” can be scored. There is a maximum of 10 points to be achieved; a score above six is considered as high quality, 4–5 as fair and ≤ 3 as poor quality. The methodological quality analysis of all included RCTs is reported in Table 2.

### Data extraction

The data extracted for each included RCT consisted of name of author, year of publication, country, type of intervention and control group, participant characteristics, applied diagnostic criteria, treatment duration, report on adverse events and treatment withdrawal, outcome measures and result/conclusion. The data extracted is summarized in Tables 3, 4, 5.

### Classification of diagnostic criteria

The diagnostic criteria were grouped according to the extent to which the importance of PEM and other core symptoms, including pain, sleep disturbances, cognitive impairment and neuroendocrine dysfunction is emphasized [52]; CF: “No PEM” (PEM is not mentioned as a criterion), CFS: “Optional PEM” (PEM is included as an optional or minor criterion) or ME: “required PEM” (PEM is a required or main criterion). The categorization of the diagnostic criteria sets is presented in Table 1.

**Table 1:** Diagnostic criteria classification with respect to the significance of PEM and other core symptoms

Criteria set ME/CFS		Post-exertional malaise inclusion		
	Author/institution	No PEM (CF)	Optional PEM (CFS)	Required PEM (ME)
CDC (1988), Holmes definition	Holmes 1988 [53], Centers for Disease Control and Prevention, USA		X	
Oxford (1991)	Sharpe 1991 [22]	X		
CDC (1994), Fukuda definition	Fukuda 1994 [23], Centers for Disease Control and Prevention		X	
London (1994)	The National Task Force on CFS/PVFS/ME 1994 [54]		X^a^	
CCC (2003), Canadian Consensus Criteria for ME/CFS	Carruthers 2003 [24]			X
Empirical CDC (2005)	Reeves 2005 [55], Centers for Disease Control and Prevention, USA		X	
NICE guideline (2007)	Nice 2007 [56], UK’s National Institute for Health and Clinical Excellence		X^b^	
Canada-revised (2010)	Jason 2010 [57]			X
ME-ICC (2011), International Consensus Criteria for ME	Carruthers 2011 [25]			X^c^
IOM (2015) (SEID)	Institute of Medicine 2015 [1]			X

^a^Requires exercise-induced fatigue, but does not require presence of all core symptoms;

^b^No PEM required (may be post-exertional fatigue) and does not require presence of all core symptoms;

^c^Post-exertional neuroimmune exhaustion (PENE)

### Data analysis

Data analysis was stratified by the three defined subgroups according to the status of PEM in the diagnostic criteria applied in the RCTs. Outcomes measured within one month of the end of treatment were regarded as post-treatment measurements (post). Follow-up assessed one to six months after treatment was considered short-term effects (short) and measurements more than six months following treatment were viewed as long-term effects (long). The statistical significance (p < 0.05) of intervention effects was reported with emphasis on fatigue and physical functioning. Results are described for each diagnostic category. For each subgroup, weighted mean values of both the Chalder Fatigue Scale (CFQ) and the physical functioning subscale of the Short Form 36-item health survey (SF-36-PF) were calculated from the available before- and after-treatment scores (preferably at short-term follow-up or the nearest measure moment).

Substantial changes (much or very much worse/better) in patient-reported global impression of change (PGIC) score were reported (PGIC−/+) as well. Conclusively, it was considered whether the findings were relevant for ME patients.

## Results

### Description of included studies

#### Study selection

The search, with applied filters, initially resulted in 239 articles. Eighteen met all eligibility criteria and were included. Three protocols, two additional effect evaluations and four additional follow-up articles linked to included RCTs were evaluated as well. The selection process for inclusion/exclusion of studies in this review is presented in a PRISMA flow diagram (Fig. 1).

The majority of the studies were conducted in Europe; six studies in the UK [58–63], two in Belgium [64, 65], and one each in The Netherlands [66], Norway [67] and Spain [68]. Four studies were performed in Asia: three in Hong Kong/China [69–71], one in Japan [72]. Additionally, one RCT was conducted in each of the countries of Australia [73], New Zealand [74] and USA [75]. Tables 3, 4 and 5 summarize the characteristics and results of the included RCTs.

#### Patient characteristics and diagnostic criteria

The study samples varied between 30 and 641 participants, with a total of 2320 participants. All participants were diagnosed with CF, ME or CFS with one of the mentioned criteria sets. Mean age varied from 34 to 48 years across the studies and 78% of the participants were women.

Three studies [58–60] used the Oxford criteria in which PEM is not included, 14 studies [61–66, 68–75] used the CDC-94/Fukuda criteria that consider PEM as an optional symptom [23], and one study [63] used the NICE criteria that requires PEM or post-exertional fatigue, but not all other core symptoms. One study used the Canadian Consensus Criteria, the only study that applied a diagnostic criteria set requiring PEM and other core symptoms.

The largest study, the PACE trial [58], applied the Oxford criteria and reported that 67% of the participants met the empirical CDC (optional-PEM) and 51% the London ME criteria (modified version) as well. In the FINE trial [59] 31% of the participants, who all fulfilled the Oxford criteria, met the London criteria as well.

#### Intervention characteristics

The therapeutic applications evaluated in this review and considered relevant for physiotherapy consisted of one or more of the following elements: physical activity, body awareness, health education or orthostatic training.

The main physical activity interventions were GET and activity pacing (AP). GET is based on the notion that the fatigue is maintained by deconditioning and avoidance of activity. Accordingly, it is assumed that one can overcome the fatigue by increasing the activity level and physical fitness by means of low-level aerobic exercise with a rigid gradual increase of intensity and amount. In some studies, heart rate monitors were used during exercise sessions to help participants meet the prescribed intensity levels [58, 61, 73, 74]. GET was given alone [58, 74] or as part of a rehabilitation program [59, 68, 76]. AP is a strategy aimed at reducing the frequency and severity of PEM by focusing on awareness and knowledge of one’s limits and early signs of exacerbation. It targets on prioritizing of activities, being as active as possible within one’s limits, and alternating active and rest periods [77]. In some programs focusing on AP [67, 75], the principles of the Energy Envelope Theory [78] were applied. According to this theory, ME/CFS patients should not expend more energy than they perceive they have (energy-envelope), as this results in PEM and increased disability. In another program [58], adapted pacing therapy (APT) was applied to encourage participants to restrict their activity levels to below 70% of their perceived limits. AP was given alone as a therapy [58], as part of GET with pacing [73], as graded exercise self-help (GES) guided by symptoms [63], as part of a rehabilitation [61], educational [75] or self-help program [63, 64, 67], or as a comparison intervention [65]. Body awareness incorporates coordinated body posture and movement, breathing, and meditation techniques. Two original eastern approaches of exercise and healing techniques, Qigong [69–71] and isometric yoga [72], were evaluated. In addition, body awareness therapy was included in a rehabilitation program [66]. Several health education programs with different objectives were included. They aimed at encouraging GET [60] or AP [75], focused on pain physiology [65] with the intention to alter pain cognitions and thereby reduce catastrophizing and kinesiophobia, or provided self-management education aimed at accepting and improving ability to cope with ME [67]. In one study, orthostatic (tilt) training was used to reduce orthostatic intolerance [62].

The control interventions consisted of care as usual [58–60, 63, 67, 74], waitlist for intervention [69–72], relaxation therapy [61, 64, 73], exercise [65, 68], CBT [58, 76], sham-training [62] or supportive listening [59]. One of the RCTs included CBT [58] and one supportive listening [59] as additional experimental arms; these were considered as control interventions in this review.

The median treatment duration was 12 weeks. It was not always clear by whom the intervention was delivered, but all interventions were considered relevant for physiotherapy despite the fact that some were led in cooperation with or by peers [67, 75], a nurse [59], an occupational therapist [58, 64, 67], a clinician therapist [60], an exercise physiologist [58, 73], a yoga instructor [72], a qigong master [69–71] or an interdisciplinary team [66, 68].

#### Outcome measures

Outcomes were mainly measured by patient-reported outcome measures (PROMs). Almost all studies had included outcome measures on fatigue and physical functioning, some on mental functioning, sleep, illness beliefs, pain and global impression of change. A total of 30 different PROM tools were applied. Most RCTs applied multiple primary outcome measures.

The 36-item Short Form Health Survey (SF-36) or the shorter forms, assessing physical and mental health status and resulting impact on everyday life (labelled here as ‘health status’), were most frequently used (69%). Some studies evaluated only single domains, usually Physical Functioning (PF). Other frequently used outcome measures included the Chalder Fatigue Questionnaire/Scale (CFQ) (56%), Hospital Anxiety and Depression Scale (HADS) (44%) and Checklist Individual Strength (CIS) (25%). Two studies [58, 72] reported on PEM occurrence. Seven studies included a PGIC score.

Seven studies reported on objective outcome measures: employment loss [79], activity monitoring [66, 73], walking ability [58], fitness [58], cardiopulmonary exercise testing [73, 74], blood biomarkers, hemodynamic and autonomic parameters [62]. One study reported autonomic function indices and blood biomarkers solely in the intervention group [80].

#### Methodological quality of the studies

Two studies [81, 82] were excluded because of poor quality. All included studies scored high (n = 15) or fair (n = 4) on the PEDro scale; PEDro scores ranged from five to eight with six as a median (Table 2). Only Sutcliffe [62] reported successful participant blinding by offering sham training to the control group. For all other interventions, it seemed unfeasible to allow participants and therapist blinding.

**Table 2:** Methodological evaluation of the included RCTs (PEDro scale [51])

Author, year	Random allocation	Concealed allocation	Groups similar at baseline	Participant blinding	Therapist blinding	Assessor blinding	Adequate follow-up < 15% missing	Intention- to- treat analysis	Between-group differences	Point estimate and variability	Total (0–10)
Chan et al. 2013 [70]	Yes	No	Yes	No	No	No	Yes	Yes	Yes	Yes	5–fair
Chan et al. 2014 [69]	Yes	No	Yes	No	No	No	Yes	Yes	Yes	Yes	6–high
Clark et al. 2017 [63]	Yes	No	Yes	No	No	No	Yes	Yes	Yes	Yes	6–high
Ho et al. 2012 [71]	Yes	No	Yes	No	No	Yes	No	Yes	Yes	Yes	6–high
Kos et al. 2015 [64]	Yes	Yes	Yes	No	No	Yes	No	No	Yes	Yes	6–high
Meeus et al. 2010 [65]	Yes	Yes	Yes	No	No	Yes	Yes	No	Yes	Yes	7–high
Moss-Morris et al. 2005 [74]	Yes	Yes	Yes	No	No	Yes	Yes	Yes	Yes	Yes	8–high
Nùñez et al. 2011 [68]	Yes	Yes	Yes	No	No	Yes	Yes	No	Yes	Yes	7–high
Oka et al. 2014 [72]	Yes	No	Yes	No	No	No	Yes	No	Yes	Yes	5–fair
Pinxsterhuis et al. 2017 [67]	Yes	No	Yes	No	No	Yes	Yes	No	Yes	Yes	6–high
Powell et al. 2001 [60]	Yes	Yes	Yes	No	No	No	Yes	Yes	Yes	Yes	7–high
Sutcliffe et al. 2010 [62]	Yes	Yes	Yes	Yes	No	No	Yes	No	Yes	Yes	7–high
Taylor et al. 2004 [75]	Yes	Yes	Yes	No	No	Yes	Yes	Yes	Yes	Yes	8–high
Thomas et al. 2008 [61]	Yes	Yes	No	No	No	Yes	No	No	Yes	Yes	5–fair
Vos-Vromans et al. 2016 [66]	Yes	Yes	Yes	No	No	No	Yes	Yes	Yes	Yes	7–high
Wallman et al. 2004 [73]	Yes	No	Yes	No	No	No	Yes	No	Yes	Yes	5–fair
Wearden et al. 2010 [59]	Yes	Yes	Yes	No	No	No	Yes	Yes	Yes	Yes	7–high
White et al. 2011 [58]	Yes	Yes	Yes	No	No	No	Yes	No	Yes	Yes	6–high

PEDro score: fair 4–5, high 6–10

Seven studies measured effects of the intervention at long-term follow-up, after one year or longer.

### Synthesis of results in view of the significance of PEM

Tables 3, 4 and 5

**Table 3:** Included RCTs with diagnostic-inclusion criteria without PEM as a criterion (CF patients)

Author, Year Country	Intervention (I)	Comparison (C)	Participants details (I/C) Number allocated (N), Mean age (year) Gender (% female)	Diagnostic criteria	Duration Session duration Frequency No. of sessions (ss), period (# weeks)	Outcome measure moments^a^ (weeks)	Main outcome measures 1 Primary 2 Secondary	Adverse events Treatment withdrawn (I/C) ITT^b^	Results (benefits), compared to control^c^ Concl.—Authors own conclusion
“PACE-trial” White et al. 2011, 2013 [58, 85] (2007 [86]) Bourke et al. 2014 [120] Sharpe et al. 2015 [121] Chalder et al. 2015 [122] McCrone et al. 2012 [79] UK	GET or APT, each in addition to SMC	SMC SMC + CBT (not evaluated here)	N = 641 (160,160/161) Age: 38 76–80%	Oxford (51% London criteria^d^, 67% empirical CDC)	GET: 14 ss, 23 weeks APT: 14 ss, 23 weeks SMC 3 ss, 52 weeks	12 rand (mid-therapy) 24 (= post) 52 rand 134 (104–230) rand	1: CFQ, SF-36-PF 2: WSAS, HADS, JSQ, PGIC, CFS symptoms, pain, fibromyalgia, PEM occurrence and poor concentration or memory, EQ-5D, 6-min walking ability, self-paced step test of fitness, lost employment	Yes [83] 24/15/17 No	Post: CFQ: GET signif, APT ns; SF-36-PF: GET signif, APT ns Long-1 year: CFQ: GET p < 0.01, APT ns; SF-36-PF: GET p < 0.01, APT ns WSAS/JSQ/HADS/PGIC: GET p < 0.05, APT ns; PGIC−/ + : GET 6/41%, APT: 7/31%. Concentration and memory: ns; PEM occurrence, pain, fibromyalgia: GET p < 0.05, ATP ns 6-min walking: GET p < .001/ns, APT ns; Fitness, lost employment, EQ-5D: GET, APT ns Serious adverse events were infrequent, non-serious adverse events were common, physical deterioration occurred most often after APT, p < 0.001 Long-2 year CFQ: GET, APT ns, SF-36-PF: GET, APT ns Concl: 1 year: GET can safely be added to SMC to moderately improve outcomes for chronic fatigue syndrome, but APT is not an effective addition. GET was more effective in reducing the frequency of both muscle and joint pain than APT and SMC, but small effect sizes 2 year: There was little evidence on long-term differences between groups
“FINE-trial” Wearden et al. 2010 [59] UK	PR—Pragmatic rehabilitation (≈CBT + GET)	GP-TAU SL-Supportive listening, general treatment	N = 296 (95/101/100) Age: 45 78%	Oxford (London criteria: 30%/31%/33%)	10 ss 18 weeks	20 basel 70 basel	CFQ, SF-36-PF, HADS, JSQ	Yes 18/17 Yes	Short: CFQ, HADS-depr, Jenkins, p < 0.05, SF-36-PF ns Long: all variables ns, No adverse events Concl: Pragmatic rehabilitation improved sleep, fatigue and depression in CFS patients, but has no long-term effect
Powell et al., 2001, 2004 [60, 123] UK	Education to encourage GET 1. Minimum intervention 2. Min. + telephone 3. Min. + face to face treatment	TAU (medical assessment, information, advice booklet, encouraging activity and positive thinking)—delayed onset (1 year)	N = 148 (37/39/38/34) Age: 34/32/33/34 78%	Oxford	1: 3 h, 2 ss 2: + 30 min, 7 tel ss 3: + 1 h, 7 ss, 12 weeks	12 rand 26 rand 52 rand 104 rand	1: SF-36-PF, CFQ 2: HADS, JSQ, PGIC	No 5,7,7/2 Yes	Long-1 year: CFQ, SF-36, HADS, JSQ: p < 0.001, 56% fulfilled no longer CFS trial criteria. PGIC−/ + : –/78% Long-2 year: benefit sustained, 56% fulfilled no longer CFS trial criteria Difference between intervention groups ns Intervention resulted in substantial improvement compared with TAU. Benefits sustained until 2 year follow-up. Delayed treatment was associated with lower efficacy

*Ss*: sessions:* ns* non-significant,* APT*: Adaptive Pacing Therapy;* CBT*: Cognitive Behavioural Therapy;* GET*: Graded exercise therapy;* TAU*: Treatment As Usual;* SMC*: Specialist Medical Care,* CFQ*: Chalder Fatigue scale/Questionnaire;* EQ-5D*: Euroqol Questionnaire;* HADS*: Hospital Anxiety and Depression Scale;* JSQ*: 4-item Jenkins Sleep Questionnaire;* PGIC*: Patient Global Impression of Change; PGICdet/impr: PGIC (very) much worse/better;* SF-36–PF*: Short Form Health Survey - Physical Functioning;* WSAS*: Work and Social Adjustment Scale

^a^Rand: from randomisation moment, basel.: from baseline, post: (at) post-treatement

^b^Data for at least one key outcome was analyzed by ‘intention to treat’ analysis (ITT)

^c^Results in favour of intervention. If results favours comparison intervention, ‘[C]’ is added. Post: post-treatment, Short-time follow-up, Long-longtime follow-up

^d^‘Second-version’, with unknown modifications

**Table 4:** Included RCTs with diagnostic-inclusion criteria with PEM as an optional criterion (CFS patients)

Author, Year Country	Intervention (I)	Comparison (C)	Participants details (I/C) Number allocated (N), Mean age (year) Gender (% female)	Diagnostic criteria	Duration Session duration Frequency No. of sessions (ss), period (# weeks)	Outcome measure moments^a^ (weeks)	Main outcome measures 1 Primary 2 Secondary	Adverse events Treatment withdrawn (I/C) ITT^b^	Results (benefits), compared to control^c^ Concl.—Authors own conclusion
Kos et al. 2015 [64] Belgium	Activity pacing self-management (APSM)	Relaxation therapy	N = 33 (16/17) Age: 41 100%	CDC-94/Fukuda	60–90 min weekly 3 weeks	Post	COPM, CIS, SF-36, CFS symptom list	No 1/3 No	Post: CIS p < 0.05, SF-36-PF and other scores ns No adverse events ASPM is feasible and effective in increasing participation in daily life activities and decreasing fatigue in women with CFS
Taylor et al. 2004 [75] USA	Immediate program group: peer-based, education, including activity pacing using the envelope theory	Delayed program group	N = 47 (23/24) Age:47 91%/100%	CDC-94/Fukuda	8 Biweekly group sessions over 16 weeks + 7 months peer counseling	52, from baseline	CFSSRF (incl CFQ), QoL index	No ? Yes	Long: Time × condition interaction: QoL, CFSSRF-Symptom severity: p < 0.05, CFQ. not reported, other items ns Concl.: Patient driven programs like this can have a positive effect on symptom severity and QoL over time in CFS/ME
Meeus et al. 2010 [65] Belgium	Pain physiology education	Pacing and self-management education	N = 48 (24/24) Age: 40 92%/75%	CDC-94/Fukuda	Once 30 min education	Post	1: NPT 2: PCI, PCS, TSK, pressure pain threshold	No None No	Post: NPT p < 0.001, PCS-rumination, -worry, -distraction p < 0.05, other PCI-scales, TSK, Pain thresholds ns Pain education results in better understanding of pain and less catastrophizing at short term
Wallman et al. 2004 [73] Australia	GET with pacing	Flexibility and relaxation	N = 68 (34/34) Age: 43 77%	CDC-94/Fukuda	max 30 min/ss 3–4 ss/week 12 weeks	4 post	PGIC, HADS, CFQ, Activity levels, Stroop test, physiological assessments (HR, BP, VO_2,_RER, net blood lactate production), RPE OAESI	No 2/5 No	Post: CFQ, HADS-depr, physiological assessments resting (except diastolic BP) and exercise, Stroop test p < 0.05/ns, HADS-anx, RPE, PGIC−/ + r: 0/60%, activity level: ns Concl: GET was associated with improvements in physical work capacity, as well as in specific psychological and cognitive variables
Thomas et al. 2008 [61] UK	Multi-convergent therapy (MCT), including CBT, GET and pacing	Relaxation therapy (+ non-randomized control)	N = 31 (17/14) Age: 48/45 71%	CDC-94/Fukuda	10 1 h ss, 10 weeks	Post 26	1: Karnofsky performance scale 2: PGIC overall, function, fatigue	Yes 5/0 No	Post: Karnofsky (83% vs 21% normal score, consultant-rated), PGIC overall p < 0.001, PGIC fatigue p < 0.001, PGIC function p < 0.05 Short: PGIC p < 0.001, PGIC fatigue p < 0.001, PGIC function p < 0.05 No adverse events Concl: MCT seemed more effective than relax therapy
Vos-Vromans et al 2016 [66] (2012 [76]) The Netherlands	MRT (CBT, gradual reactivation, body awareness therapy, pacing, social reintegration)	CBT	N = 122 (60/62) Age: 40 80%	CDC-94/Fukuda criteria	CBT: 45–60 min, 16 ss, 6 months MRT: 33 h, 10 weeks	26 basel (post) 52 basel	1: CIS-f, SF-36 2: SES, SCL-90, MAAS, SIP-8, CAL, LSQ, EET, PSCG, activity monitor	Yes, but no reported 6/12 Yes	Short: CIS: ns, SF-36-MCS ns, SF-36-PCS ns, SES p < 0.05, PSCG < 0.001, all others ns Long: CIS p < 0.05, SF-36-MCS ns, SF-36-PCS ns, SES p = 0.01, PSCG < 0.001, EET < 0.05 all others ns Concl: MRT is more effective than CBT in reducing long-term fatigue severity in CFS
Clark et al 2017 [63], (2016 [124]) UK	Guided graded exercise self-help (GES) and four guidance sessions with physiotherapist	SMC	N = 211 (97/102) Age: 38 82%/76%	NICE (71% CDC-94 and 81% Oxford)	8 weeks	12 rand 52 rand	1: SF-36-PF, CFQ 2: PGIC-health adverse outcomes, PGIC, HADS, PHQ-13, WSAS, IPAQ,	Yes 29%/– Yes	Short: CFQ p < 0.001; SF-36-PF p < 0.01, WSAS p < 0.05, HADS p < 0.01, IPAQ p < 0.001, PHQ-13, PGIC−/+ : 0/14%, ns Long: not reported (yet?) No serious adverse reactions, serious deterioration: ns Concl: GES is a safe intervention that might reduce fatigue and, to a lesser extent, physical disability for CFS
Moss-Morris et al. 2005 [74] New Zealand	GET	SMC	N = 49 (25/24) Age: 41 71%	CDC-94/Fukuda	12 weeks	Post 42 basel	PGIC, CFQ, SF-36-PF, VO_2_ peak (treadmill), IPQ-R, IMQ	No 3/– Yes	Post: PGIC−/ + : –/56% p < 0.05, CFQ: p < 0.05, IMQ-symptom focus p < 0.05 SF-36-PF, VO_2_ peak, IPQ-R: ns Short: Phys. CFQ < 0.05, mental CFQ, SF-36-PF ns Concl: GET appears to be an effective treatment for CFS and it operates in part by reducing the degree to which patients focus on their symptoms
Nùñez et al. 2011 [68] Spain	MRT (group CBT- and GET, and conventional pharmacological treatment)	Exercise counselling (and conventional pharmacological treatment)	N = 120 (60/60) Age: 43 93% / 14%	CDC-94/Fukuda criteria	CBT: 90 min, 9 ss, GET: 60 min, 9 ss 10–12 weeks	52 post	1: SF-36 2: HAQ, HADS, FIS	SF-36 reduced 2/3 No	Long: SF-36-BP: (C) (p < 0.05), other results ns Concl: MRT was not superior to usual treatment at 12 months in terms of HRQL (SF-36). The combination of GET and CBT is ineffective and might be harmful to some patients
Sutcliffe et al. 2010 [62] UK	Home orthostatic (tilt) training (HOT)	Sham training	N = 38 (19/19) Age: 48 84% / 79%	CDC-94/Fukuda	40 min. 26 weeks	4 rand (mid treatment) Post	1: Compliance/tolerability 2: FIS, hemodynamic parameters	Yes, but not reported 5/5 No	Short: FIS: ns, systolic blood pressure drop with active stand p < 0.05, other hemodynamics ns Concl: HOT is well tolerated and generally complied with
Oka et al. 2014 [72] Japan	Isometric sitting yoga (and pharmacotherapy)	Waitlist (pharmacotherapy alone)	N = 30 (15/15) Age: 38 80%	CDC-94/Fukuda	20 min 5.8x/week, 9.2 weeks (mean 5.6ss with instruction)	Post 8	POMS (F and V), CFQ, SF-8, occurrence	Yes None No	Post: POMS-F p < 0.001, POMS-V p < 0.01 Short: CFS p < 0.01, SF-8 total ns. Absence of serious adverse events or PEM Concl: Isometric yoga reduced fatigue and improved vigor
Chan et al. 2014 [69] Hong Kong	Qigong (Baduanjin)	Waitlist	N = 150 (75/75) Age: 39 46%/62%	CDC-94/Fukuda	16 lessons of 1.5 h over 9 weeks	Post 12 post	PSQI, CFQ, HADS, PGIC	Yes 4–49/– Yes	Short: PSQL: ns, CFQ: p < 0.001, HADS < 0.05/ < 0.001. PGIC−/ + : –/68%. Except muscle ache, adverse events were uncommon Concl: Qigong was an efficacious and acceptable treatment for sleep disturbance in CFS
Chan et al. 2013 [70] Hong Kong	Qigong	Waitlist	N = 154 (77/77) Age: 42 72% / 82%)	CDC-94/Fukuda	2 times/week 5 weeks + 12 weeks home-based practice	Post	CFQ, HADS	Yes 5/12 Yes	Post: CFQ, HADS p < 0.001 No adverse events were reported Concl: Qigong may not only reduce the fatigue symptoms, but also has antidepressive effect
Ho et al. 2012 [71] Hong Kong	Qigong	Waitlist	N = 70 (35/35) Age = 42 76% / 84%	CDC-94/Fukuda	5 weeks group qigong + 12 weeks home-based practice	Post	CFQ, SF-12	Yes 8/10 Yes	Post: SF-12-PF: ns CFQ, SF-36 MF: p < 0.001 No adverse effects were reported Concl: Qigong exercise may improve CFS and mental functioning

Ss: sessions: ns non-significant, CBT: Cognitive Behavioural Therapy; GET: Graded exercise therapy; MRT: Multidisciplinary Rehabilitation Treatment; SMC: Specialist Medical Care, BP: blood pressure; CAL: Causal Attribution List; CFQ: Chalder Fatigue scale/Questionnaire; CFSSRF: Chronic Fatigue Syndrome Symptom Rating Form; CIS/CIS-f: Checklist Individual Strength – Fatigue severity subscale; COMP: Canadian Occupational Performance Measure; EET: Improvement and Satisfaction questionnaire; EQ-5D: Euroqol Questionnaire; FIS: Fatigue Impact Scale; HADS: Hospital Anxiety and Depression Scale; HAQ: Health Assessment Questionnaire; HR: heart rate; IMQ: Illness Management Questionnaire: IPQ: Illness-Perception Questionnaire; LSQ: Life Satisfaction Questionnaire; MAAS: Mindfulness Attention Awareness Scale; NPT: Neurophysiology of Pain Test; OAESI: Older Adult Exercise Status Inventory; PAQ: International Physical Activity Questionnaire; PCI: Pain Coping Inventory; PCS: Pain Catastrophizing Scale; PGIC: Patient Global Impression of Change; PGICdet/impr: PGIC (very) much worse/better; PHQ-13: Patient Health Questionnaire-13; POMS: Profile of Mood States, Fatigue and Vigor; PSCG: Patient-Specific Complaints and Goals questionnaire; RPE: Ratings of perceived exertion; RER: respiratory exchange ratio; SF-8/12/36: Short Form Health Survey (–PF physical functioning, -MF mental functioning - BP: Bodily Pain, -PCS: physical component summary, -MCS mental component summary); SCL-90: Symptom Check List-90; SES: Self-Efficacy Scale; SIP-8: Sickness Impact Profile, 8 items; TSK: Tampa Scale of Kinesiophobia; VO2 oxygen uptake; WSAS: Work and Social Adjustment Scale

^a^Rand: from randomisation moment, basel.: from baseline, post: (at) post-treatement

^b^Data for at least one key outcome was analyzed by ‘intention to treat’ analysis (ITT)

^c^Results in favour of intervention. If results favours comparison intervention, ‘[C]’ is added. Post: post-treatment, Short-time follow-up, Long-longtime follow-up

^d^‘Second-version’, with unknown modifications

**Table 5:** Included RCTs with diagnostic inclusion criteria with PEM as a required criterion (ME patients)

Author, Year Country	Intervention (I)	Comparison (C)	Participants details (I/C) Number allocated (N), Mean age (year) Gender (% female)	Diagnostic criteria	Duration Session duration Frequency No. of sessions (ss), period (# weeks)	Outcome measure moments^a^ (weeks)	Main outcome measures 1 Primary 2 Secondary	Adverse events Treatment withdrawn (I/C) ITT^b^	Results (benefits), compared to control^c^ Concl.—authors own conclusion
Pinxsterhuis et al 2017 [67] Norway	Group-based self-management education, based on a self-efficacy theory and the ‘energy envelope’ theory (pacing)	CAU	N = 146 (73/73) Age: 44 94% / 82%	CCC and CDC-94/Fukuda criteria	2.5 h every 2 weeks, 16 weeks	26 52	1: SF-36 2: FSS, SES, ICQ	No 2/6 No	Short: SF-36 ns, FSS (C) p < 0.05, SES p < 0.05, ICQ ns Long: all outcome ns Concl.: this self-management program for CFS patients did not show a sustained effect

Ss: sessions: ns non-significant, CAU: Care As Usual, FSS: Fatigue Severity Scale; ICQ: Illness-Cognition Questionnaire; SES: Self-Efficacy Scale; SF-36: Short Form Health Survey

^a^Rand: from randomisation moment, basel.: from baseline, post: (at) post-treatement

^b^Data for at least one key outcome was analyzed by ‘intention to treat’ analysis (ITT)

^c^Results in favour of intervention. If results favours comparison intervention, ‘[C]’ is added. Post: post-treatment, Short-time follow-up, Long-longtime follow-up

^d^‘Second-version’, with unknown modifications

#### RCTs with diagnostic inclusion criteria without PEM as a criterion

All three RCTs (Table 3) showed effectiveness of GET or GET-encouraging interventions on post- or short-term fatigue and mental health in CF patients. Effects might sustain until 1-year follow-up. Effect on physical function was significant following GET and education. APT did not seem effective. Long-term effects on mental fatigue and physical function are unclear. In the PACE study, both GET and APT were not able to reduce employment loss or increase fitness [58, 79]. Improvements on the walking test were greater for the GET group than for the control [58]. However, improvements and group differences were small and all results were still just over half of normal values.

The intervention groups’ mean CFQ scores (11-item version, 2 RCTs) were 28.4 at pre, 22.7 at post, and SF-36-FP (3 RCTs) were 34.6 at pre, 46.2 at post.

#### RCTs with diagnostic inclusion criteria with PEM as an optional criterion

In CFS patients (Table 4), it was unsure whether GET improved fatigue and mental health, while effect on physical functioning was absent or negative. AP, GET with pacing, qigong and yoga seemed effective in reducing post-treatment and short-term fatigue. Effects on health status and physical functioning, in particular, were unlikely while effects on mental health and physiological parameters were unsure.

In the intervention group, the mean CFQ scores (14-item version, 6 RCTs) were 28.7 at pre, 18.4 at post, and SF-36-FP (7 RCTs) were 41.8 at pre, 46.7 at post.

#### RCTs with diagnostic inclusion criteria with PEM as a required criterion

One RCT evaluated an intervention for ME patients (Table 5). Pinxsterhuis 2017 [67] compared group-based self-management to care as usual. The program focused on AP and illness coping and was effective at short-term follow-up for fatigue and self-efficacy. There were no significant differences between the groups with regard to physical functioning. The program for ME patients did not show long-term effects.

In the intervention group, the mean SF-36-FP scores were 48.1 at pre, 46.5 at post.

#### Adverse events and compliance

Ten studies mentioned the occurrence of adverse events. Two of the GET studies in CF patients reported on adverse events. The PACE study devoted an entire paper on this subject [83]. The conclusion was that the numbers of adverse events did not differ significantly between trial treatments (GET 8%, APT 9%), but physical deterioration occurred most often after APT (GET 11%, APT 25%). No adverse events were reported following pragmatic rehabilitation [59]. The two GET studies in CFS patients (Nùñez [68] and Moss-Morris [74]) did not evaluate adverse events. However, in the discussion of Nùñez it was mentioned that the intervention might have been harmful for some participants due to a significant pain increase (SF-36-BP). In addition, Moss-Morris [74] mentioned that the physiological assessment tests were experienced as harmful to more than 50% of the participants. Two studies on GET with pacing evaluated adverse events in CFS patients. No adverse events were found following multi-convergent therapy [61]. In the GES-trial [63], serious adverse events were uncommon, but in the guided graded exercise self-help group, as well as in the control group, about a quarter of participants reported deterioration of physical functioning (reduction of SF-36-PF score of 10 points). The four RCTs on qigong [69–71] or yoga [72] reported that adverse events were either not seen or uncommon, except for some muscle ache. In addition, it was explicitly mentioned that none of the participants reported PEM after practicing yoga [72]. The orthostatic training also seemed to be well tolerated [62]. In the RCT with ME patients, adverse events were not evaluated [67].

Compliance with the activity protocols was seldom directly evaluated. In the PACE trial, however, ‘adequate treatment’ (participation in ≥ 10 of the 14 sessions) was reported: 85% for GET and 90% for APT (ns). In the GES trial [63], the physiotherapists reported that 42% of the participants adhered to GES completely or very well, 30% moderately well, and 29% slightly or not at all. Vos-Vromans [66] reported that all participants in the MRT group and 88% in the CBT group reached the 70% level of compliance to treatment. In one of the Qigong trials [70], it was reported that 25% had completed < 9 sessions and 32% had completed all 16 sessions.

## Discussion

The main aim of this literature review was to appraise the effect of physiotherapeutic interventions on symptoms and functioning of patients with ME/CFS, in view of the significance of PEM in the applied diagnostic criteria. The intention was thereby to contribute to improving recommendations for evidence-based physiotherapeutic care for the ME/CFS patients with PEM.

Many researchers and health professionals fail to acknowledge ME as a distinct clinical entity. Accordingly, the labels CFS and ME are often used synonymously in both research and clinical practice. Also, patients that obtained a CF label in this review are frequently labeled as CFS elsewhere and CFS patients may be categorized as ME patients. Therefore, all relevant RCTs with ME/CFS patients that investigated the effect of an intervention considered relevant for physiotherapy were analyzed. In order to establish the potential benefit or possible harm of the studied interventions, the RCTs were synthesized narratively in terms of the applied diagnostic criteria for inclusion, the results, the focus on possible adverse events, and the conclusions.

### Summary of main results

This review found indications that GET was moderately effective, possibly until 1-year follow-up, in reducing fatigue for CF patients diagnosed with the broad Oxford criteria. In CFS patients, mainly diagnosed with the Fukuda criteria, several interventions, including GET, GET-encouraging interventions, GET with adaptive pacing, qigong and yoga seemed moderately effective in reducing fatigue, though only at post-treatment. The interventions might also have been effective in improving physical functioning in CF patients, but not in CFS patients. However, effects, if any, vanished when evaluating objective outcomes; no convincing effects were obtained in fitness, level of physical activity, employment, etc. AP appeared not to be effective in CF, though possibly effective for post-treatment fatigue reduction in CFS. Only one RCT for ME patients experiencing PEM was identified [67]. Unfortunately, the self-management and AP education program evaluated in this RCT seemed ineffective. Thus, one cannot draw conclusions on the effect of applied physiotherapeutic interventions to date for this patient group*.* The shortage of trials evaluating effectiveness of interventions in ME patients is not specifically related to the physiotherapy field, as it has been seen in pharmacological, psychological and behavioral interventions as well [33, 44, 84].

### Methodological considerations of the included studies

There are some methodological inadequacies in the included RCTs concerning method of diagnosis, choice of outcome measures, selective reporting and heterogeneity of the samples.

In the majority of the studies it was not clear how the ME/CFS diagnosis was set; following a prior thorough clinical examination or solely using self-reported symptoms. Some trials that applied wide criteria had incorporated more narrow criteria for subgroup analyses. The PACE [58] and the FINE trials [59] evaluated London criteria (CFS-criteria) in addition to the Oxford criteria and found that a considerable subgroup fulfilled both criteria. The PACE trial assessed fulfillment of the empirical CDC CFS criteria as well. Contrary to expectation, diagnostic subgroup analysis in the PACE trial showed comparable treatment effects on fatigue and physical functioning. However, the correctness of these diagnoses is uncertain as the evaluation of symptoms of these additional diagnostic criteria covered only the last week, and not the previous six months as defined in the criteria sets [85]. Another critical point is that adverse events in these subgroups were not evaluated. For the FINE trial, subgroup analyses were not reported at all.

Concurring with the inclusion criteria, all included articles were graded as ‘high’ or ‘fair’ quality according to their PEDro score. Maximum achieved score was 8 out of 10, as blinding of subjects and therapists seems unfeasible in most physiotherapeutic practice. Despite a comprehensive design and protocol, well-powered and with a high-quality score, the most extensive and influential RCT, the PACE trial [58, 86], has been heavily criticized [87]. Besides criticism for using the broad Oxford criteria, it has been denounced for protocol changes of effectiveness. Re-analysis demonstrated that most of the modest improvements did not reach the level of significance in the GET group when compared to the control group [88]. Another critical issue is the absence of long-term follow-up results and lack of group differences in the objective outcome measures, which were more or less ignored in the reporting.

Although several ME/CFS symptoms can be assessed using well accepted objective testing methods [89, 90], the conclusions of the evaluated RCTs were primarily based on subjective PROMs. Remarkably, the clinical relevance of the achieved improvements was rarely discussed. Fatigue and physical functioning were most frequently evaluated. The occurrence of PEM was assessed as an outcome measure in only two RCTs [58, 72]. Its operationalization was unclear in both studies and remarkable in one study, where several participants reported PEM at baseline and did not fulfil the 1994 London criteria [54] that requires post-exertional fatigue. To evaluate changes in PEM, interventions towards ME should report on several specific aspects of PEM; not only the occurrence of PEM, but also perceived severity. Assessments of changes in presence, frequency, and intensity of various PEM symptoms, time aspects and trigger intensities would be valuable. A couple of PROMs [11, 91–93] and objective tests [89] are available to evaluate PEM. Apart from fatigue, other core symptoms were usually not evaluated either. In contrast, in many RCTs, depression and anxiety symptoms were evaluated as an outcome measure. This seems to be a paradox since, in most diagnostic criteria sets, psychiatric conditions are listed as an exclusion criterion.

With ME/CFS, even if the participants improved on average, it is of particular interest to know how many participants experienced negative changes and to what degree. Selective reporting of patient-reported impression of change scores made it difficult to evaluate this. Seven RCTs included a PGIC score, but only three studies reported both the portions that experienced substantially negative and positive change [58, 63, 73].

Thirteen of the 18 RCTs applied the CFQ. However, comparing results among different interventions and diagnostic subgroups appears difficult as two different versions (11 and 14 items) and two scoring methods (bimodal and Likert scale) were applied. Nonetheless, it is clear that changes were modest. Further, the after-treatment CFQ values (mean approx. 22) were still far above normal (approx. 14 [94]) and long-term effects were mostly lacking. Another important issue is that CFQ appears inappropriate to evaluate changes in fatigue in ME/CFS patients because of ceiling effects. The high mean inclusion scores indicate that most of the participants had reported a maximum score (“much more than usual”) on most items at pretest. Consequently, it is impossible to rate any exacerbations, only potential improvements. This was pointed out earlier by Morris as well [95].

Improvements on the physical function subscale of SF-12/36 were not as frequent as for fatigue; SF-PF was only significant in two of the three CF trials and in one out of 6 CFS trials. However, similar to the CFQ, improved SF-36 scores at follow-up (approx. 47 on average) were still far below normative data (approx. 90 in the corresponding age group, 35–54 years) and even below the norm of age group 75–84 years, which averages a mean SF-36-PF score of 58 [96].

Even though most interventions involved physical activity aspects, only one third of the RCTs’ applied objective outcome measures to assess physiological or functional capacity changes. A few studies obtained statistically significant improvements on these measures, but they were often hardly or not clinically relevant. Like the subjective measures, values were considerably below normal values for (sedentary) healthy people. This clearly indicates lasting reduced physical functional capacity in patients with ME/CFS [97]. Aside from Wallman (2004 [73]), who assessed cognitive function with a modified Stroop color test, objective assessments of other aspects of neurocognitive functioning or other dimensions like PEM, lack of energy, muscle function or sleep impairment were lacking.

CF populations and, to a lesser extent, CFS populations may comprise a mixed group of fatigued patients with or without PEM. This may complicate appropriate adjustment of intensity of physical activity instructions for all participants. PACE’s APT instructed the participants to do 30% less than their available energy might allow [98]. It has been suggested that if the expended energy was consistently lower than available energy, as instructed in APT, participants both with and without PEM, might have become too inactive, resulting in reduced physical and mental functioning and increased social isolation instead [77]. This may have been problematic, especially for the 33% of participants in the PACE study [58] who had a depressive disorder (and probably not ME). Even so, absence of convincing objective improvements following GET at group level may suggest that the level of intensity may not have been appropriate for everyone. As depressed participants and other chronic fatigued patient groups often tolerate exercise well, they may accordingly achieve improved physical capacity [99, 100]. Such patients were probably included in the CF populations. An important issue, however, is that it seemed that the main problem in ME patients is their reduced ability to adapt and recover from exercise or exertion intolerance, in general, rather than deconditioning or reduced exertion capacity itself [106]. GET intensity may have been too high for the ME/CFS patients with PEM, causing deteriorations and non-compliance. This may have reduced average improvements on the group level. Non-compliance was demonstrated in a GET-like case–control study in which daily activity was assessed by an accelerometer [101]. Initially, the ME/CFS patients were able to reach the prescribed activity goals, however, after 4–10 days, they seemed unable to sustain target activity levels and reported pronounced worsening of symptoms. Repeated testing or monitoring over time may therefore give more relevant data than just one single exertion test.

Several GET studies applied heart rate monitoring to guide training intensity and to reduce participants’ focus on bodily symptoms. Unfortunately, no RCT included in this review evaluated these or other objective measures to report on compliance with the exercise regime. Potential associations with the measured PROMs were generally not reported on either. However, a recent study reported positive correlations between objectively measured and patient reported physical functioning (SF-PH) in ME/CFS patients [102]. Continuing low SF-PH scores, as seen in this review, may therefore confirm the impression that the participants’ level of physical activity did not notably increase following the interventions.

From this review, it seems that proven effectiveness of physical exercising in ME/CFS is associated with the subjectivity of the applied outcome measures. PROMs that evaluate subjective experiences of fatigue more frequently obtained statistically significant differences than scoring of self-perceived limitations to perform specific physical activities, as in SF-36. Further analyses of data from PACE and two other CBT studies illustrated that effect sizes increased when the subjectivity of the outcome measure increased [103]. This was also reported in re-analyses of the Cochrane review on exercise therapy for ME/CFS. This review based its conclusions on PROMs only and suggested that exercise therapy likely has a positive effect on fatigue [35]. Analyses of the objective outcomes of the included RCTs, however, demonstrated that GET does not lead to clinically significant objective improvements [104]. Using PROMs only may therefore be incorrect in ME/CFS research. This is consistent with findings of clear discrepancies between what is measured in research and patients’ reported perception in a systematic review of PROMs in ME/CFS research [105].

Besides the reduced effectiveness with diminished outcome objectivity, physiotherapeutic treatment effectivity also seems to disappear when follow-up time or diagnostic specificity increases. Unfortunately, this leaves us with little evidence when it comes to effective physiotherapeutic management of ME patients.

### Current evidence concerning potential negative responses to treatment

From the studies in this review, no clear and direct indication was found regarding participants’ tolerability of the interventions. Few studies reported on the occurrence of adverse events or non-adherence due to intolerance to the intervention. However, in intervention research involving ME/CFS patients with PEM, reporting of adverse effects seems of particular significance [106]; interventions are not necessarily harmless when adverse effects and compliance not have been systematically reported.

Furthermore, absence of substantial mean improvements on PROMs and objective measurements may indicate that some participants have improved, while other participants may have worsened on these measures. PGIC scores confirm that not all participants perceived substantial improvement following the interventions; across the RCTs, 22% to 86% rated their change from very much worse to a little better. Only one included GET study with CFS patients commented on the harmfulness of the intervention [68].

Clear indications of potential negative patient-reported experiences of common ME/CFS interventions are summarized in a review of 11 patient surveys [107]. More than half (55%) of the ME/CFS patients undertaking GET (n = 4876) reported negative outcomes and only 27% reported a decrease of symptom severity. In contrast, pacing (n = 8981) obtained the lowest negative response rate (4%) and the highest reported benefit (81%).

The concerns regarding exercise programs are confirmed in several case control studies evaluating responses on sub-maximal activity in ME/CFS patients. Adverse responses have been found as disproportional increases of fatigue, sleep disturbances and pain, as well as disturbances in muscular, neuroimmunological and cognitive functioning [1, 2, 19, 108, 109]. These adverse responses are supported by evidence of exercise-induced maladaptive findings across multiple systems during or after maximal or submaximal physical activity. Deviations have been reported, for instance, in brain activation, immune and autonomic response, pain modulation, lowered aerobic metabolism and metabolic deficits [1, 2, 15–21]. Several of these alterations are correlated with the perceived intensity of PEM [12, 109, 110]. Although we mainly focused on physical exertion here, this largely applies to cognitive, sensory and psychological stressors as well.

Even though the results of this review did not reveal substantial negative responses, the marginal and doubtful effects, patient-reported experiences and evidence coming from biomedical research strongly suggest an overall reduction in tolerance of physical exertion in ME patients.

### Strength and limitations of this review

A strength of this review is that the included RCTs reported on a broad specter of outcome measures, interventions and aim of treatment. A limitation was the heterogeneity of comparison groups, group sizes and follow-up duration. This heterogeneity limited the possibility to compare results and calculate effect sizes across the different treatments and diagnostic groups. Therefore, a narrative synthesis was conducted.

This systematic review was limited by deficiencies of the trials. Several of these have already been described in the methodology overview. One of the objectives was to evaluate and discuss the reported physiotherapeutic interventions in view of (potential) harm and adverse effects for patients with ME. A limitation of this current review was therefore the lack of focus in the RCTs on participants’ tolerability of the interventions and modest reporting of possible adverse effects.

To improve evidence quality, searches were restricted to RCTs published since the year 2000. We may thereby have missed additional valuable knowledge concerning other relevant interventions that were only reported in weak methodological RCTs or non-randomized trials. Although the first criteria set that required PEM, the Canadian Consensus Criteria, was published in 2003, we found only one RCT that studied ME patients, which is an insufficient basis for assessing the effects of physiotherapy. Remarkably, these newer diagnostic criteria are still rarely used in intervention studies. This was observed in a recent systematic review covering 55 RCTs targeting ME/CFS as well [33].

As far as we know, this is the first available review that stratified synthesized evidence of ME/CFS RCTs according to significance of PEM in the inclusion criteria. However, several previous systematic reviews mentioned some uncertainty as to whether findings in studies with Oxford or Fukuda criteria are applicable to ME/CFS patients diagnosed with criteria requiring PEM [33, 35, 37, 44]. In an update to an evidence report concerning ME/CFS [84], any evidence of beneficial effect for GET disappeared by excluding the trials using Oxford criteria for inclusion. In contrast, a recent review restricting their search to European RCTs that applied diagnostic criteria excluding mental health illnesses (7 Fukuda, 1 CCC) [43] concluded that effects of rehabilitation and activity pacing were inconsistent and comparable to previous systematic reviews that had included Oxford criteria. The newly published systematic review of Ahmed (2020 [44]) had a partly similar intention as this current review, but was restricted to CBT and GET interventions. All RCTs included Oxford or Fukuda criteria only. They could not find evidence to conclude that CBT and GET are effective treatments for CFS patients.

We are aware of one earlier systematic review on the effect of physiotherapy in ME/CFS patients [34]. It focused on RCTs published 2007–2017 and included four studies. Two of these studies were excluded in our review because they either involved a younger population or were listed as a separate RCT [111] while actually reporting secondary analyses of an already included RCT [66]. In this current review a much broader spectrum of physiotherapeutic interventions for ME/CFS patients is included. We have, indeed, included some interventions that were guided by other health care professionals but nonetheless considered relevant for physiotherapy. Still, we do not fully understand why the other 12 RCTs we found in the same period were not included in the systematic review of Galeoto 2018 [34]. They might have had a dissimilar view of what may be applied as a physiotherapeutic intervention.

In the literature, CBT interventions have been predominant in published RCTs targeting ME/CFS [33]. Although it has been suggested that other health care professionals than clinical psychologists may deliver CBT-based treatment strategies [112], CBT studies were not included in this review. CBT usually addresses possible depressive symptoms, maladaptive thoughts and illness beliefs that may impair recovery. In ME/CFS, CBT often applies a graded increase in physical activity strategies as well. In physiotherapy, however, the rationalization to apply GET may rather be to reverse a cycle of inactivity and deconditioning. There may be other interventions of debatable relevance that were excluded in this review as they were assumed to fall outside the general skillset of a physiotherapist; acupuncture is one example we are aware of.

The majority (78%) of the participants were women, which is in accordance with the general ME/CFS patient population that has a female-to-male ratio ranging from 2:1 to 5:1 [113]. Inclusion of severely affected patients, however, did not seem representative. In general, 10 to 25% of the ME/CFS population is severely or very severely affected and house- or bedbound [114]. In the included studies, most participants were apparently mildly or moderately affected. This was expected, as severely affected patients are often unable to participate in trials if attendance to a healthcare facility is required. It is therefore doubtful whether the findings of this review are applicable for severely affected patients. However, including them in experimental RCTs seems unethical. In agreement with Mengshoel (2020 [43]), we underline the need for case studies and patient experiences to develop meaningful physiotherapeutic management procedures for this group of patients as well.

Classification of the diagnostic criteria sets was not entirely unambiguous. Although the focus was primarily on the presence of PEM, other core symptoms were emphasized as well. This is in line with a diagnostic algorithm described by the US Institute of Medicine (IOM, 2015 [1]) that required the presence of PEM, unrefreshing sleep, cognitive impairment and orthostatic intolerance. The NICE [56] and London criteria [54] both mention the inclusion of PEM-like symptoms (exercised induced fatigue and post-exertional fatigue), but do not require the presence of all core symptoms and were therefore classified in the CFS cluster.

### Implications for clinical practice

Expanding knowledge concerning effects and possible consequences of physiotherapy in ME patients seems necessary [1, 115]. In general, the prescription of a physical activity program is common and considered beneficial in physiotherapeutic practice [116]. Contrary to most conditions, however, it seems that even briefly increased physical activity may cause abnormal detrimental responses in ME patients [108, 117]. Unfortunately, knowledge of ME/CFS, and PEM in particular, still seems insufficient among physiotherapists. This is in line with findings of a recent survey among ME patients [32]; around half of the respondents had received physiotherapy, but a worrying 53% of them reported that physiotherapy made their ME symptoms worse.

Many researchers and health professionals still fail to acknowledge that subgroups of ME/CFS require different management approaches, which can have serious adverse consequences for ME patients [118]. A few European countries have national guidelines for treatment of ME/CFS [47]. Their recommendations are solely based on (weak) evidence from RCTs with CF or CFS patients, but are extrapolated to ME patients as well. This brings up an unusual aspect of external validity in the translation to clinical practice; the study samples in ME/CFS research usually seem to be more heterogenic than those of the more distinct ME population. GET, together with CBT, is still the treatment most often recommended in the European guidelines [47]. In USA, however, the CDC removed GET and CBT as recommended treatments in 2017.

For physiotherapists, it is important to take into account the diagnostic criteria used in research and recommendations when translating this evidence into practice and selecting appropriate therapy for ME patients. This also applies to the patients with greater disease severity. Although some of the concepts of the discussed interventions in this review may be applicable, the interventions themselves are potentially harmful for patients with severe or very severe ME.

As the present review shows, there is currently no scientific evidence for curative or beneficial treatment for ME. It is essential to acknowledge this and refrain from applying potentially harmful treatments. Here, one needs to rely even more on patient and clinician experience and evidence from biomedical research. Physiotherapeutic management should focus on symptom relief and increasing or maintaining health status and quality of life, by improving the ability to cope with ME, guiding self-management and avoiding PEM, in particular. With this in mind, health education, pacing and body awareness can be valuable approaches.

With the current public health situation, these approaches may also be very relevant for recovered COVID-19 patients who experience persistence of symptoms [49]. Here, it is important for physiotherapists to understand that ME may be a potential complication of a viral infection [119] and that standard care may be detrimental for these patients.

This review focused on physiotherapeutic interventions only. It is not to be expected that physiotherapy may cure ME, but it may contribute to symptom relief, coping and maintenance or improvement of functioning. In addition to this, and hopefully to find a cure for ME, we need to lean on biomedical research and future effective pharmacological therapy.

### Implication for future research

To generate strategies for effective treatment, further understanding of the pathophysiological bases of the disease is essential [6]. This review documents important knowledge gaps about the consequence of the presence of PEM on physiotherapeutic management of ME patients. It identifies a critical need for consensus to apply updated diagnostic criteria in future diagnosing, interventional and biomedical research to further understanding of ME. At present, the CCC or updated ME-ICC seem to be the most obvious alternatives for this. Generally, it is important to differentiate between CF, CFS and ME. Without this clear distinction between patients with or without PEM, it is unfeasible to provide health care providers with evidence of the most adequate treatment. ME is a complex condition with an extensive clinical heterogeneity. Therefore, even if proper diagnostic criteria are used, it is important to apply subgroup, predictor and moderator analyses to attain better targeted therapeutic options.

As diagnosis is still based on patient-reported symptoms, inclusion of PROMs in ME/CFS research is obvious. These PROMs need to cover several core symptoms of ME, including PEM, and must be capable of assessing both improvements and deteriorations in symptoms and functioning. In ME/CFS it is of particular relevance to report the proportion of participants that may experience exacerbation of symptoms and not only average changes for the study population. Further, one also has to ascertain whether PROM changes are associated with objectively measured changes and are clinically meaningful. From a clinical point of view, it is relevant to ensure adequate length of follow-up and to report and evaluate harms, other adverse effects, adherence and reasons for withdrawal.

## Conclusion

Currently, there is no scientific evidence when it comes to effective physiotherapy treatment for ME patients diagnosed with narrow diagnostic criteria sets that include PEM. Findings indicating effectiveness of physiotherapeutic interventions for ME/CFS are mainly based on RCTs involving patients diagnosed with diagnostic criteria that do not require PEM. Possible evidence vanished when diagnostic specificity, outcome objectivity or follow-up time increased.

As any exertion may cause long-lasting exacerbation of symptoms in ME patients, some interventions may have adverse consequences. Hence, in the translation of ME/CFS research evidence to clinical practice, it is crucial to differentiate between patients diagnosed by criteria with or without PEM as a required feature.

To improve evidence, well-defined ME populations, reporting of adverse effects, sufficient follow-up and incorporation of relevant and objective measures are essential in interventional research.

## Data Availability

The articles reviewed in this study are available in the public domain.

## Abbreviations

AP: Activity pacing
APT: Adapted pacing therapy
CBT: Cognitive behavioral therapy
CCC: Canadian consensus criteria
CDC: Centers for Disease Control and Prevention, USA
CF: Chronic fatigue
CFQ: Chalder fatigue scale
CFS: Chronic fatigue syndrome
GET: Graded exercise therapy
IOM: Institute of Medicine
MCT: Multi-convergent therapy
ME: Myalgic encephalomyelitis
ME-ICC: International consensus criteria
MRT: Multidisciplinary rehabilitation treatment
ns: Non-significant
PEM: Post-exertional malaise
PGIC: Patient global impression of change
PGIC−/+: Substantial changes on PGIC: (very) much worse/better
PROM: Patient-reported outcome measures
RCT: Randomized controlled trial
SEID: Systemic exertion intolerance disease
SF-12/36: Short form 12/36-item health survey
SF-36-PF/BP/PH: SF-36-physical functioning/ bodily pain/ physical health subscale
